# Progress in Bacteriology

**Published:** 1900-01-13

**Authors:** 


### Progress in Bacteriology..

**The Fusiform Bacillus.**—Vincent[1] describes this organism as associated with a pseudo-membranous tonsilitis resembling diphtheria. There is fever, adenitis, and difficulty in swallowing, and beneath the membrane a superficial ulceration. The pharyngeal membrane is red and œdematous. In children the disease is very fatal. The bacillus is fusiform in shape, 6-12$\mu$ in length, may be straight or curved. It stains readily with the aniline dyes, and involution forms show non-staining vacuoles. It does not stain with Gram's method. It is best demonstrated by preparing a film by rubbing a piece of the membrane on a cover-slip. The bacilli are then seen to be present in great numbers, accompanied by a spirillum. Neither of these organisms have as yet been cultivated on artificial media, nor have they been shown to be pathogenic to animals. The fusiform bacillus is a normal inhabitant of the buccal cavity.

**The Plague Bacillus.**—Stewart[2] gives the following tests for the plague bacillus. If grown on the surface of dry gelatine or agar plates small colourless transparent colonies with ragged edges are seen in 24-48 hours. Later the colour of the colony changes to that of mother-of-pearl, and appears heaped up in the centre. The colonies slip about on the surface of the agar when touched by the wire. Agar tubes inoculated with broth cultures present a "ground glass" appearance. If a drop of oil be added to a broth tube, and the whole sterilised, and then inoculated with a plague culture, a characteristic stalactitic growth is obtained. The bacillus stains with the aniline dyes, but not with Gram. Bipolar nodules are common. The organism is short, and is frequently found in pairs or short chains. In pulmonary cases of plague the bacillus is present in the sputum, and it may also be obtained from a non-suppurating bubo by making an incision over the gland and drawing off fluid in a sterilised capillary glass pipette. It is found in the blood shortly before death.

**Influenza Bacillus.**—An early diagnosis of influenza may, according to Wynekoop,[3] be made by the microscopical examination of films made from rubbings taken from the tonsils and pharynx of persons suffering from the disease. The bacilli are present in large numbers, sometimes solitary, sometimes in short chains. They stain well with carbol fuchsin, and may be distinguished from the pneumococcus by their smaller size, more blunted ends, and lesser affinity to the stain. The influenza bacillus is seldom found in the blood. It grows well on serum, especially if hæmoglobin be added, appearing in from ten to fifteen hours as a clear transparent colony. In bouillon it forms a sediment only, and on no medium does it live longer than a few days. The influenza bacillus may occur in conjunction with B. diphtheriæ, and may be the determining cause in certain cases of acute conjunctivitis.

**Bacteria in Rheumatic States.**—Wohlmann[4] reviews the specific nature of the organisms described in acute rheumatism and in rheumatoid arthritis. In acute rheumatism cocci have been observed in the blood by many workers. In 1891 Achalme found in the heart blood and cerebro-spinal fluid a bacillus resembling that of anthrax. It stained with Gram, but best with feebly alkaline methylene-blue. Glycerinated horse-bouillon was the best medium for growth, which is strictly anaërobic. Spores are formed. It readily grows in the urine of arthritic subjects, and cultures have a marked sour smell, and are checked by addition of dilute salicylic acid. Achalme's bacillus is associated with cocci in many cases. It has been found to excite endocarditis and other rheumatic sequelæ in guinea pigs when injected subcutaneously. These results have been confirmed by a large number of other observers. With regard to rheumatoid arthritis, the organism isolated from joint fluids by Wohlmann, Bannatyne, and Blaxall still holds the field. It is a bacillus with marked polar staining, $2\mu \times \cdot 75\mu$, decolorised by Gram's method, and best stained by very weak methylene blue. It grows on peptone beef broth as minute, gold, floating colonies, and on agar as a grey film. Their observations have apparently been confirmed by Chauffard and Ramon. Schüller has, however, found in the synovial fluid of rheumatoid joints a bacillus $2\cdot 6\mu \times \cdot 75\mu$ staining easily with fuchsin and aniline dyes, growing readily on agar and liquefying gelatine in from two to six days. So far no inoculation experiments with Bannatyne's bacillus have been worked out.

**Bacteriology of the Conjunctiva.**—Lawson[5] tested the normal conjunctiva of 200 persons for micro-organisms. Of these 41 were sterile, 30 showed staphylo-or pneumococci, 118 the xerosis bacillus (90 in pure culture), 13 bacilli (including 2 of the mesentericus group), 6 sarcinæ, and 1 torulæ. Those of the cocci isolated were pathogenic to rabbits, their virulence being much reduced by sojourn in the sac. The xerosis bacillus must be regarded as a normal inhabitant of the conjunctiva. It is probable that the lachrymal secretion has an inhibitory effect upon the growth and life of micro-organisms. In the discussion following Lawson's paper, Eyre stated that he found that one-half of the sacs inspected were sterile. He had found, however, virulent staphylo- and strepto-cocci in some cases. Eyre[6] describes a condition of conjunctivitis excited by a diplo-bacillus. The disease (angular conjunctivitis) is most common in middle-aged women, and is contagious. The symptoms consist in photophobia, lachrymation, with greyish mucous discharge, and injection of conjunctiva. Its course is very chronic. In film preparations the bacilli are seen arranged in pairs or short chains. It grows on serum agar or serum (with liquefaction), is a non-motile, non-sporing aërobic bacillus, $3\mu \times 1\mu$. In films from cultures it resembles a diplococcus. There is no capsule. Involution forms are common. It stains well with all aniline dyes, but is decolorised by Gram's method. A culture inoculated into a healthy human conjunctiva sac induces an attack of the disease.

Morax and Petit[7] point out that Weeks's bacillus, a diplo-bacillus (*vide* supra), and the gonococcus can penetrate the tissues and directly excite conjunctivitis; the cocci, on the other hand, only invade when the surface is wounded; whilst the diphtheria bacillus is always a secondary invader. Acute contagious conjunctivitis, such as is frequently epidemic in schools, is generally due to Weeks's bacillus, rarely to pneumococcus. Angular or subacute conjunctivitis is most frequently associated with the diplo-bacillus. The pneumococcus is often present in the normal sac; occasionally its virulence may become increased, and give rise to conjunctivitis. If pneumococci are introduced from without the infection is far more virulent, and the disease contagious. The following description of Weeks's bacillus is given by Neustatter[8]: A small, fine bacillus, with pointed ends, and a characteristic gap in the middle, found loose in the discharges, or in groups in the cell; staining with analine dyes, decolorised by Gram; does not grow well on ordinary media.

Conjunctivitis may also be excited by the ozæna bacillus, the micrococcus conjunctivæ minutissimus, staphylococcus (especially when the duct is involved), streptococcus, and B. influenzæ. The nature of the infection cannot be judged from clinical examination alone.

[1] Annal. de l'Inst. Tast., Aug., 1899. [2] Brit. Med. Jour., Sept. 16, 1899. [3] Med. News, Mar. 11, 1899. [4] Brit. Med. Jour., Nov. 11, 1899. [5] Brit. Med. Jour., Aug. 20, 1899. [6] Ibid. [7] Annales d'Oculist, Sept., 1898. [8] Treatment, Feb. 23, 1899.

